# Clinical Significance of Composition and Functional Diversity of the Vaginal Microbiome in Recurrent Vaginitis

**DOI:** 10.3389/fmicb.2022.851670

**Published:** 2022-02-18

**Authors:** Min Jeong Kim, Seungok Lee, Mi Yeon Kwon, Myungshin Kim

**Affiliations:** ^1^Department of Obstetrics and Gynecology, Bucheon St. Mary’s Hospital, College of Medicine, The Catholic University of Korea, Seoul, South Korea; ^2^Department of Laboratory Medicine, Incheon St. Mary’s Hospital, College of Medicine, The Catholic University of Korea, Seoul, South Korea; ^3^Department of Clinical Medicine Research, Bucheon St. Mary’s Hospital, The Catholic University of Korea, Seoul, South Korea; ^4^Department of Laboratory Medicine, Seoul St. Mary’s Hospital, College of Medicine, The Catholic University of Korea, Seoul, South Korea

**Keywords:** recurrent vaginitis, vagina, taxonomy, microbiome, *Lactobacillus* spp.

## Abstract

**Objective:**

The vaginal microbiome protects the female genital tract from various diseases, such as vaginitis, a vaginal inflammation characterized by abnormal discharge, itching, and pain. To evaluate the clinical relationship between the vaginal microbiome and the pathophysiology of recurrent vaginitis (RV), we investigated the microbiome taxonomic profile (MTP) in the vaginal samples of Korean female patients with RV.

**Methods:**

Forty women of reproductive age diagnosed with RV were enrolled. The vaginal MTP of patients was analyzed using 16S ribosomal RNA gene sequencing, and the results were compared with that of healthy women (*n* = 100). Further, the association of the vaginal community state type (CST) with the clinical characteristics was analyzed.

**Results:**

The species abundance of MTP was significantly lower in patients with RV than in healthy women (*p* < 0.05), whereas species evenness and diversity were significantly higher in patients with RV than in healthy individuals (*p* < 0.05). The proportion of the most common vaginal *Lactobacillus* spp. was significantly lower in the MTP of patients with RV than healthy women (*p* < 0.01). The beta diversity distance was also significantly different between patients with RV patients and healthy individuals (*p* = 0.001). Based on the CST, the MTP of 40 RV samples was categorized as follows: 21 (52.5%) for CST IV, 8 (20.0%) for CST III, 5 (12.5%) for CST I, 2 (5.0%) for CST II, 1 for (2.5%) for CST V, and 3 (7.5%) for mixed CST. Patients with underlying uterine diseases (uterine leiomyoma, adenomyosis, and endometrial polyps; *n* = 17) showed higher species richness and diversity than those without (*n* = 23; *p* < 0.05).

**Conclusion:**

Changes in the species abundance and microbial diversity in the vagina were strongly associated with RV. A low proportion of *Lactobacillus* spp. was found in patients with RV than in healthy women. The abundance and diversity of bacterial taxa were significantly higher in patients with underlying gynecologic disease than those without. Our study offers an insight into the nature of the vaginal microbiome and proposes that surveying the vaginal microbiome is valuable for detecting and treating gynecologic diseases in the future.

## Introduction

Vaginitis is a vaginal mucosal inflammation characterized by abnormal vaginal discharge, malodor, irritation, itching, or burning. Many women who have vaginitis go undiagnosed or have recurrences after treatment. In addition, patients have to visit various hospitals regularly due to recurring symptoms. In some patients, identifying disease-causing microorganisms using bacterial culture and molecular techniques can help determine the course of treatment; however, it cannot always prevent disease recurrence.

Studies have shown that the vaginal microbiome is closely associated with various gynecological diseases (leiomyoma, adenomyosis, and endometriosis; Green et al., 2015, Chen et al., 2017). Vaginal dysbiosis, or the long-term alteration of the vaginal microbiome, is generally caused by the loss of dominance of the *Lactobacillus* spp. (van de Wijgert and Jespers, 2017). Vaginal dysbiosis induces the overgrowth of anaerobic and facultative bacteria. It facilitates other reproductive tract disorders, such as desquamative inflammatory vaginitis, which has been linked with poor pregnancy outcomes, pelvic inflammatory disease, and increased risk of sexually transmitted infections (Cohen et al., 2012; Caretto et al., 2017).

Next-generation sequencing (NGS) methods, such as high-throughput 16S rRNA gene sequencing, have been used to investigate the vaginal microbiome as they can analyze the relative abundance of community microorganisms (Shipitsyna et al., 2013). It can also determine the composition and diversity of the vaginal microbiome in the form of microbiome taxonomic profile (MTP) and community state type (CST). Thus, 16s rRNA sequencing contributes to understanding the relationship between the microbiome and clinical disorders. Several studies on vaginal microbiota have been conducted in Caucasian cohorts from the United States and Europe (Ravel et al., 2011; Huang et al., 2014), but studies involving Asian patients are rare (Lee et al., 2020).

In this study, we investigated the characteristics of the vaginal microbiome in Korean women with recurrent vaginitis (RV). NGS was performed on patient samples, and the results were compared to those of healthy women. We also categorized the CST groups based on the vaginal microbiome and evaluated their characteristics and associations with clinical conditions.

## Materials and Methods

### Study Design and Subject Enrollment

All patients who visited our hospital between October 2019 and November 2020 with RV were enrolled in the Department of Obstetrics and Gynecology Hospital. After obtaining informed consent, participants were screened to confirm their eligibility before enrollment. This study was conducted in accordance with the amended Declaration of Helsinki on the ethical conduct of research involving human subjects.

During the study period, all patients visited the obstetrician-gynecology hospital more than once with symptoms of RV. The enrolled patients ranged in age from 20 to 55 years old and were selected based on the following criteria: (a) patients who visited the hospital with vaginitis symptoms more than once in 1 year and (b) patients with apparent vaginitis symptoms (e.g., itching, vaginal discharge, and odor). The exclusion criteria were the use of chemotherapy or any antibiotics, vaginal suppositories, or consumption of hormone products 3 months before the hospital visit. Other exclusion criteria included the presence of systemic diseases (diabetes mellitus, cancer) or a history of total hysterectomy or vaginal surgery.

For comparison with healthy women (*n* = 100), the 16S rRNA gene sequencing data tagged as Project I from healthy, adult, North American females, collected from the posterior fornix and urogenital tracts, were obtained the Public Database of the Human Microbiome Project (BioProject PRJNA48333; Human Microbiome Project Consortium, 2012).

### Vaginal Sampling

Samples from the enrolled subjects were collected by a physician using a flocked swab (NFS-2 swab, Noble Biosciences, Inc., Hwaseong-si, Korea) from the posterior fornix of the vaginal wall. The collected sample was suspended in 1 ml of phosphate-buffered saline and immediately transferred to the laboratory for storage at −80°C until further use.

### DNA Extraction and 16S rRNA Gene Sequencing

For DNA extraction, a total of 40 vaginal swab samples were thawed on ice, vortexed for approximately 10 s, and centrifuged at 1,455 × *g* for 10 min. The pellets were resuspended in 100–180 μl of lysis buffer (20 mM Tris–HCl pH 8.0, 2 mM sodium EDTA, 1.2% Triton X-100, 20 mg/ml), containing 20 mg/ml lysozyme from chicken egg whites (Sigma, St. Louis, MO, United States), and incubated for 30 min at 37°C. After 30 min, proteinase K and buffer AL from the Qiagen DNeasy Blood and Tissue kit (QIAGEN, Venlo, Netherlands) were added and incubated for 30 min at 56°C according to the manufacturer’s protocol. DNA concentration and quality were measured using a spectrophotometer (Eppendorf, Hamburg, Germany). DNA concentration of more than or equal to 15 ng/μl with A_260_/A_280_ ratio of 1.8–2.0 was used for 16S rRNA sequencing.

The genomic DNA was amplified by polymerase chain reaction (PCR) using fusion primers 341F (5′-AATGATACGGCGACCACCGAGATCTACAC-XXXXXXXX-TCGTCGGCAGCGTC-AGATGTGTATAAGAGACAG-CCTACGGGNGGCWGCAG-3′) and 805R (5′-CAAGCAGAAGACGGCATACGAGAT-XXXXXXXX-GTCTCGTGGGCTCGG-AGATGTGTATAAGAGACAG-GACTACHVGGGTATCTAATCC-3′; the underlined sequence indicates the complementary region of the primer) binding to the V3–V4 regions of the 16S rRNA gene. The amplification conditions were as follows: initial denaturation at 95°C for 3 min, followed by 25 cycles of amplification (denaturation at 95°C for 30 s, primer annealing at 55°C for 30 s, and extension at 72°C for 30 s), with a final elongation at 72°C for 5 min. PCR amplification and sequencing were performed at ChunLab, Inc. (Seoul, Korea), using the Illumina MiSeq Sequencing System (Illumina, United States) according to the manufacturer’s instructions.

### Bioinformatics Analyses

The raw sequence reads were pre-processed for quality checks, such as trimming of low-quality amplicons, MTP, diversity calculation, comparative MTP, and taxonomic biomarker discovery analyses, and analyzed using the 16 s rRNA gene sequencing-based MTP of an EzBioCloud App,^1^ a ChunLab bioinformatics cloud platform (ChunLab, Inc., Seoul, Korea). Briefly, pre-processed raw sequences with low-quality (<Q25) reads were filtered using Trimmomatic ver. 0.32. After the quality check, paired-end sequence data were merged using VSEARCH version 2.13.4, followed by trimming of primers, extraction of non-specific amplicons that do not encode 16S rRNA, and detection of chimeric reads. Species-level identification was conducted using the EZBioCloud database. The Operative taxonomic unit (OTU) was picked using the open-reference method with a 97% cutoff similarity. For comparative MTP, copy number normalization (read count, gene copy number) and cutoff (%) of 1.0% for *et cetera* (ETC) were applied. MTP diversity was calculated using alpha diversity indices, such as ACE, Chao1, and Jackknife for species richness and Shannon, NPShannon, and Simpson for species diversity and evenness. Beta diversity distances were calculated to visualize the sample difference with the principal coordinate analysis (PCoA) by the Jensen–Shannon method. Beta set-significance analysis between two MTP sets was performed using the permutational multivariate analysis of variance (PERMANOVA) test. Additionally, taxonomic biomarkers were determined using the linear discriminant analysis (LDA) effect size (LEfSe) and Kruskal–Wallis H test.

### Statistical Analyses

Non-parametric comparison tests (Kruskal–Wallis test for more than two groups and Mann–Whitney U-test for two groups) were used to compare MTP results using MedCalc version 19.8 (MedCalc Software, Ostend, Belgium). Statistical significance was set at *p* < 0.05.

## Results

### Patient Demographics

Forty women were enrolled in this longitudinal study. The study participants were all Korean women, with a mean age of 44.05 ± 7.3 years and a body mass index (BMI) of 23.47 ± 3.41 kg/m^2^. The majority of women (*n* = 33, 82.5%) were premenopausal. Clinical characteristics of patients with RV are summarized and displayed using descriptive statistics in Table 1. Gynecological symptoms included vaginal discharge (*n* = 22, 55%), itching (*n* = 4, 10%), malodor (*n* = 1, 2.5%), with at least 13 patients reporting two or more symptoms (32.5%). Urological symptoms (increased frequency or urge of urination) were observed in 35 patients (87.5%). Among premenopausal women, 13 (32.5%) had irregular menstrual cycles, and eight had heavy menstrual bleeding (20%). Underlying gynecological diseases [uterine leiomyoma (*n* = 10), adenomyosis (*n* = 3), and endometrial polyps (*n* = 4)] were observed in 17 patients (42.5%).

**Table 1:** Characteristics of 40 patients with RV.

Characteristics	
Number	40
Age (years) (mean)	44.05 ± 7.3
Height (cm)	160.56 ± 4.45
Weight (kg)	60.58 ± 9.85
BMI (Kg/m^2^)	23.47 ± 3.41
Menopause (%)	7 (17.5)
Nulliparity (%)	5 (12.5)
**Gynecologic symptoms (%)**	
Vaginal discharge	22 (55)
Itching	4 (10)
Odor	1 (2.5)
Combine	13 (32.5)
**Urological symptoms (frequency, urgency %)**	
No	35 (87.5)
Yes	5 (12.5)
**Menstrual cycle (%)**	
Irregular	13 (32.5)
Regular	21 (52.5)
Unchecked	6 (15)
**Heavy menstrual bleeding (%)**	
No	25 (62.5)
Yes	8 (20)
Unchecked	7 (17.5)
**Underlying uterine diseases (leiomyoma, endometrial polyp, adenomyosis)**	
No	23 (57.5)
Yes	17 (42.5)

### Overall Sequence Output

The total valid reads from patient samples (*n* = 40) ranged from 3,277 to 79,775 (median = 33,017) after removing low-quality amplicons, non-target amplicons, and chimeras from the 16S rDNA gene sequencing results. The average read length was 420 ± 7 bp per sample. The number of OTUs ranged from 10 to 160 (median = 23), whereas the number of species ranged from 8 to 156 (median = 19) in samples obtained from 40 patients with RV.

The total number of valid reads obtained from the samples of healthy women (*n* = 100) ranged from 3,320 to 52,645 (median = 6,114). The number of OTUs found in the samples ranged from 8 to 99 (median = 26), whereas the number of species found in the samples ranged from 8 to 98 (median = 25). The overall percentage of valid reads identified at the species level in 40 patients (99.3 ± 2.7%) was similar to that of 100 healthy women (99.3 ± 0.8%).

### Significant Differences in Vaginal MTP Between Patients With RV and Healthy Women

**Figure 1:**
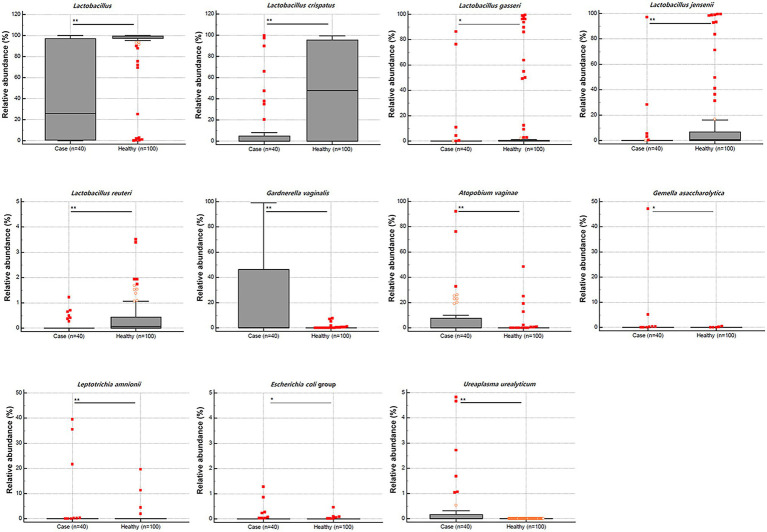
Relative abundance (%) of the statistically significant taxa (*y*-axis) between 40 cases with recurrent vaginitis (RV) and 100 healthy individuals (*x*-axis). The horizontal and box plots show the median and quartiles, respectively. Non-parametric test, ^*^p < 0.05, and ^**^p < 0.01.

We compared vaginal MTP and found that the abundance of *Lactobacillus* spp. was significantly lower in samples from patients with RV (*n* = 40) than those from healthy women (median 25.80 vs. 99.23, *p* < 0.001; Figure 1). When 24 representative microbial taxa were analyzed at the species level, the relative abundance of *Lactobacillus crispatus*, *Lactobacillus gasseri*, *Lactobacillus jensenii*, and *Lactobacillus reuteri* was significantly lower in patients with RV than in healthy women (*p* < 0.001, 0.044, <0.001, and <0.001, respectively). In contrast, the populations of *Gardnerella vaginalis*, *Atopobium vaginae*, *Gemella asaccharolytica*, *Leptotrichia amnionii*, *Escherichia coli* group, and *Ureaplasma urealyticum* were significantly higher in patients with RV than in healthy women (*p* < 0.001, *p* < 0.001, *p* = 0.005, *p* < 0.001, *p* = 0.020, and *p* < 0.001, respectively).

**Figure 2:**
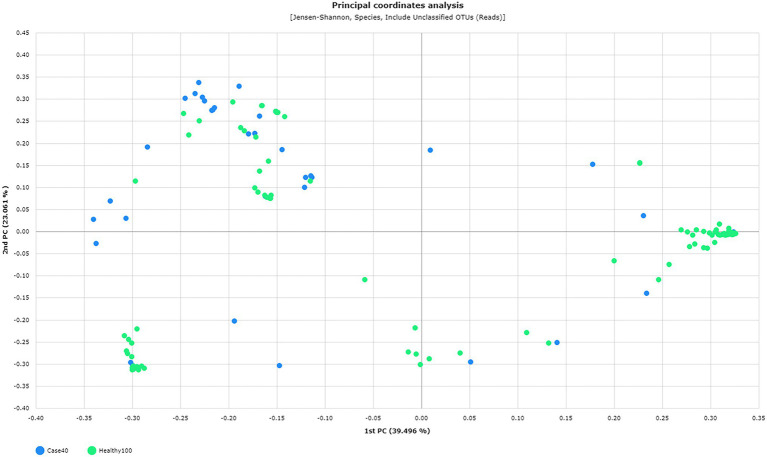
Principal Coordinate Analysis (PCoA) plots between 40 patients (green) and 100 healthy (blue) individuals based on the Jensen–Shannon beta diversity distances at the species level (*p* = 0.001, pair-wise PREMANOVA with 999 permutations).

Furthermore, the species richness, diversity, and complexity (alpha and beta diversity indices) were significantly different between the microbiota of the two groups. The species richness indices, including ACE, Chao1, and Jackknife, were significantly lower in patients with RV than in healthy women (median 24.50% vs. 40.60%, *p* = 0.011; 17.00% vs. 29.30%, *p* = 0.003; 21.00% vs. 32.00%, *p* = 0.086, respectively). In contrast, the NPShannon and Shannon diversity indices were significantly higher in patients with RV than in healthy women (median 0.68 vs. 0.33, *p* = 0.035; 0.68 vs. 0.31, *p* = 0.029, respectively), whereas the Simpson index was lower (median 0.55 vs. 0.89, *p* = 0.012) in patients with RV than in healthy women. These results indicate higher species diversity and evenness in patients with RV than in healthy women. Beta diversity distance was also significantly different between patients with RV and healthy women (*p* = 0.001, pair-wise PREMANOVA with 999 permutations). A comparative analysis of PCoA plots between RV and healthy women is outlined in Figure 2, based on the Jensen–Shannon beta diversity distances at the species level.

### Six CST Groups Based on the Dominant *Lactobacillus* spp.

Based on the *Lactobacillus* spp. dominance, we taxonomically classified 40 vaginal microbiome compositions into six CST groups: *L. crispatus*-dominant CST group I (*n* = 5, 12.5%); *L. gasseri*-dominant CST group II (*n* = 2, 5.0%); *Lactobacillus iners*-dominant CST group III (*n* = 8, 20.0%); CST group IV with low abundance of *Lactobacillus* spp. and increased diversity and abundance of anaerobic species (*n* = 21, 52.5%); *L. jensenii*-dominant CST group V (*n* = 1, 2.5%); mixed CST group with co-dominance of more than two *Lactobacillus* spp. (*n* = 3, 7.5%; De Seta et al., 2019). The detailed microbiome taxonomic composition is shown in Supplementary Table 1.

**Figure 3:**
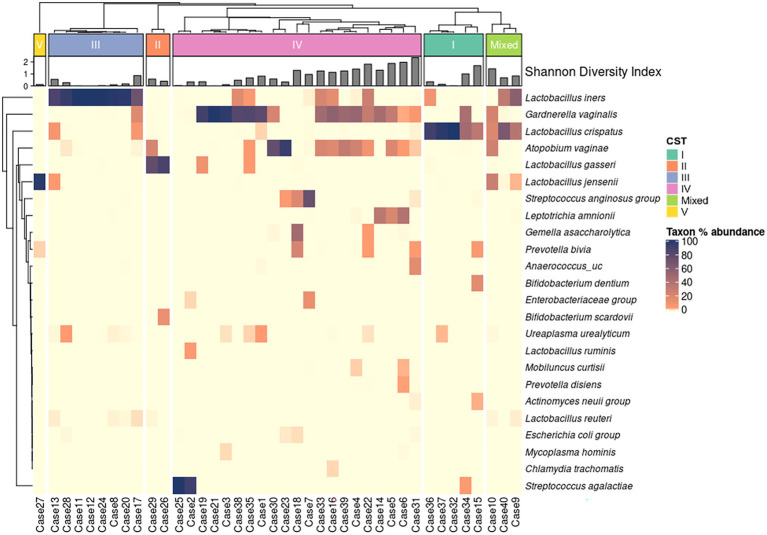
Heat map of the relative abundance of representative 24 microbial taxa in the vaginal microbiota of 40 patients with RV (color key indicating Taxon % abundance). The hierarchical (UPGMA) clustering is based on the community state types (CST) and the species composition of the vaginal microbiome. Shannon diversity indices show species diversity and evenness for each sample.

The relative abundance (%) of 24 representative microbial taxa at the species level was presented on a heatmap. Hierarchical clustering (based on unweighted pair group method with arithmetic mean, UPGMA) showed accurate clustering of the relative abundance according to the CST group (Figure 3).

**Figure 4:**
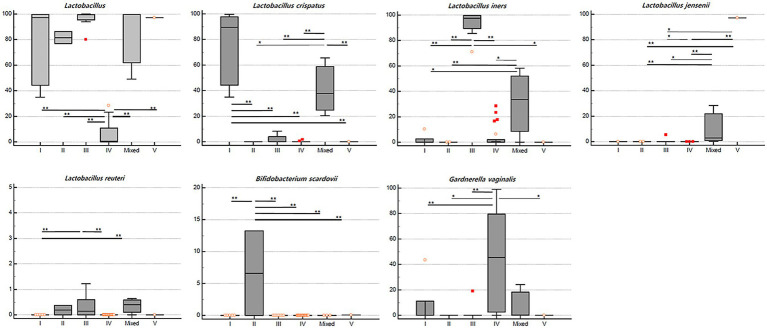
Relative abundance (%) of the significant taxa (*y*-axis) among vaginal CST (*x*-axis) in RV (*n* = 40). The horizontal and box plots showing the median and quartiles, respectively. Non-parametric test, ^*^p < 0.05, and ^**^p < 0.01.

On comparing the relative abundance of different bacterial taxa among the CST groups, we observed that the relative abundance of *Lactobacillus* spp. was significantly lower in the CST group IV than in the other CST groups (*p* < 0.01; Figure 4). The populations of *L. crispatus*, *L. iners*, *L. jensenii*, *L. reuteri*, *Bifidobacterium scardovii*, and *G. vaginalis* were also significantly different among the CST groups (*p* < 0.001, <0.001, <0.001, 0.004, <0.001, and 0.003, respectively). The statistical significance of the relative abundance of bacterial taxa between the two groups was displayed as ^*^p < 0.05 and ^**^p < 0.01 in Figure 4.

**Figure 5:**
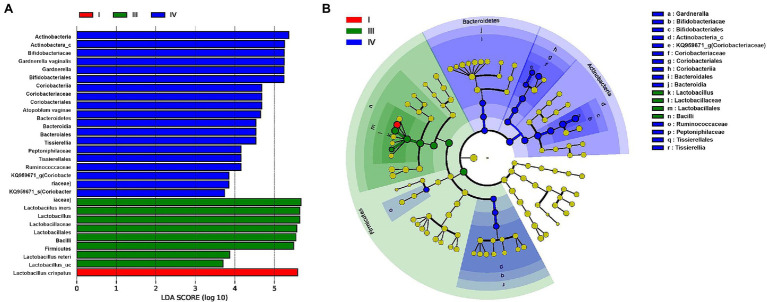
Differential abundance of the bacterial taxa in three CST (I, III, and IV) groups of vaginal samples of patients with RV **(A)** Linear discriminant analysis (LDA) effect size (LEfSe) analysis indicating significant differences in bacterial taxa (LDA score > 2.0; *p* < 0.05). **(B)** Cladogram based on LEfSe shows the phylogenic distribution of the bacterial taxa associated with CST I (red), III (green), and IV (blue) groups. Taxonomic levels of the phylum are labeled, and class, order, family, and genus are abbreviated.

The LEfSe analysis used to determine the relative abundance of bacterial taxa (LDA score > 2.0; *p* < 0.05) showed significant statistical enrichment. The phylum Bacteroidetes and lower taxa, including the order Bacteroidales, phylum Actinobacteria, and genera KQ959671_g (*Coriobacteriaceae*) and *Gardnerella*, and the phylum Firmicutes and lower taxa including family *Peptoniphilaceae* were significantly enriched in CST group IV. In contrast, the phylum Firmicutes and lower taxa, including genus *Lactobacillus* spp., were significantly enriched in CST groups I and III (Figure 5).

### Gynecological Diseases Disrupted the Vaginal Microbiome

**Figure 6:**
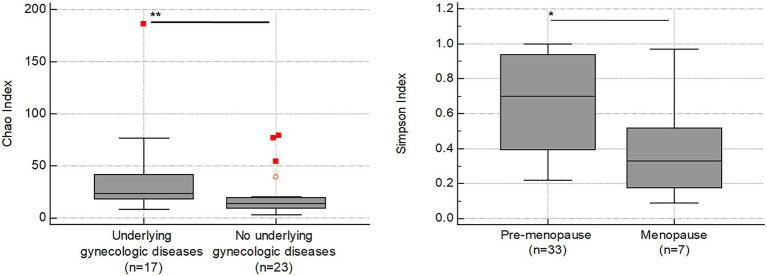
Association between alpha diversity indices (*y*-axis) and clinical symptoms (*x*-axis) in RV (*n* = 40). The horizontal and box plots show the median and quartiles, respectively. Non-parametric test, ^*^p < 0.05, and ^**^p < 0.01.

In our study, alpha diversity indices associated with species richness and diversity showed variations depending on the presence or absence of uterine diseases (Table 2; Figure 6). Patients with uterine diseases (*n* = 17) showed higher species richness indices of Chao and Jackknife (*p* = 0.006 and 0.003, respectively) and species diversity indices of Shannon, NPShannon, and Simpson (*p* = 0.035, 0.034, and 0.043, respectively) than those without diseases (*n* = 23).

**Table 2:** Association between alpha diversity indices and clinical characteristics in RV (*n* = 40, value of *p*).

Index	Min ~ Max (median) (*n* = 40)	Age 20–29y (*n* = 2) 30–39y (*n* = 3) 40–49 (*n* = 27) 50y~ (*n* = 8)	Gynecologic disease (*n* = 17)	Urologic symptom (*n* = 5)	Vaginitis symptom Discharge = (*n* = 22) Itching (*n* = 4) Odor (*n* = 1) Combine (*n* = 13)	Menstrual cycles regular (*n* = 21)	Menopause (*n* = 7)	BMI > 25 (*n* = 10)	Menorrhagia (*n* = 8)
ACE	4.11–253.02 (24.51)	>0.05	>0.05	>0.05	>0.05	>0.05	>0.05	>0.05	>0.05
Chao	3.00–185.80 (17.00)	>0.05	**0.006**	>0.05	>0.05	>0.05	>0.05	>0.05	>0.05
Jackknife	4.00–358.86 (21.00)	>0.05	**0.003**	>0.05	>0.05	>0.05	>0.05	>0.05	>0.05
NPShannon	0.01–3.12 (0.68)	>0.05	**0.034**	>0.05	>0.05	>0.05	>0.05	>0.05	>0.05
Shannon	0.01–3.05 (0.68)	>0.05	**0.035**	>0.05	>0.05	>0.05	>0.05	>0.05	>0.05
Simpson	0.09–1.00 (0.55)	>0.05	**0.043**	>0.05	>0.05	>0.05	**0.039**	>0.05	>0.05

Value of *p* was calculated by non-parametric test. Statistically significant (*p* < 0.05) was shown in bold.

We further found that the Simpson index was also lower in patients going through menopause (*n* = 7; *p* = 0.039), indicating increased species diversity in menopause status.

However, no significant association was found between the CST groups and other variables, such as uterine diseases, menopause, urological symptoms, gynecologic symptoms, menstrual cycle, menorrhagia, and BMI (data not shown).

## Discussion

Vaginitis is a condition that has a negative impact on women’s quality of life, with some women expressing anxiety, shame, and hygiene concerns, particularly those who have recurrent symptoms (Cohen et al., 2012; Smith and Ravel, 2017). A thorough analysis of the vaginal microbiota in RV may help elucidate its etiology. This study characterized the vaginal microbiota of 40 RV patients based on 16S rRNA sequencing. By comparing the MTP between patients with RV and healthy women, we found that a microbial community low in *Lactobacillus* spp. dominated the patient vaginal samples. *Lactobacillus* spp. dominance is well correlated with healthy vaginal states (Sirota et al., 2014; Aldunate et al., 2015; Garcia-Velasco et al., 2017). In agreement with previous studies, our results showed a negative association between the severity of postmenopausal atrophic vaginitis and the abundance of vaginal *Lactobacillus* spp. (Mitchell et al., 2018). Along with the relative depletion of *Lactobacillus* spp., we found the disease-causing microorganisms, such as *G. vaginalis*, *A. vaginae*, *G. asaccharolytica*, *L. amnionii*, *E. coli*, and *U. urealyticum*, were more abundant in RV. We further showed that the vaginal microbiome in patients with RV was more diverse and even. Therefore, we concluded that the changes in the predominant species in the microbial community could result in increased susceptibility to inflammation, resulting in RV.

We classified the patient’s vaginal microbiota according to the CST groups based on the dominance of *Lactobacillus* spp. (Hillier, 2005). Our results showed that CST group IV with a low abundance of *Lactobacillus* spp. but increased diversity and anaerobic population was the most common (52.5%) among patients, followed by the *L. iners*-dominant CST group III (20.0%), *L. crispatus*-dominant CST group I (12.5%), *L. gasseri*-dominant CST group II, and *L. jensenii*-dominant CST group V. These findings indicate that the lack of abundance of *Lactobacillus* spp. plays an important role in RV etiology. Interestingly, *L. crispatus*, *L. gasseri*, and *L. jensenii* typically reside as singularly predominant microorganisms in the vaginal microbiome, whereas *L. iners* is found as a member of the polymicrobial vaginal flora often transitioning to bacterial vaginosis (Muzny et al., 2018).

According to one study, there is no racial difference in the vaginal microbiome of healthy women (Beamer et al., 2017). Ravel et al. (2011) reported that vaginal CST of *Lactobacillus* (groups I, II, III, and V) were found in 80.2% and 89.7% of Asian and Caucasian women, but in only 59.6% and 61.9% of Hispanic and black women. In our study, vaginal CST of *Lactobacillus* (groups I, II, III, V, and mixed) was reported in 47.5%. Our study results differed from those of the Asian healthy group, which could be due to RV. In particular, our study is important as it investigated RV, and it will be instrumental in understanding the pathophysiology of RV and preventing recurrence.

The abundance and diversity of bacterial taxa were significantly higher in patients with gynecological diseases (uterine leiomyoma, adenomyosis, and endometrial polyps) than in those without. In a previous study, the abundance of *Lactobacillus* spp. was found to be high in the vaginal samples from patients without uterine leiomyoma, whereas patients with adenomyosis showed depleted or enriched bacterial abundance throughout the reproductive tract (Chen et al., 2017). How the disruptions to the vagino-uterine microbiota might eventually lead to benign or malignant conditions threatening women’s health is still a major concern, and further studies are required to address it.

The association between the clinical symptoms of RV (itching, vaginal discharge) and the occurrence of CST groups was difficult to establish in this study. This may be due to the small number of participants (40) enrolled in our study that prevented an effective evaluation of the relationship between the clinical symptoms and the composition of vaginal microorganisms. It may also be because symptom classification was highly subjective in this study, as objective indicators were insufficient.

The monitoring of the RV microbiome has been deferred because the primary goal of this study was to conduct comprehensive profiling of the microbiome in RV patients. However, five out of the 40 patients revisited our clinic due to recurring symptoms after the first visit. As a result, additional research comparing the vaginal microbiome at initial and follow-up visits is required.

In recent years, the health benefits of probiotic supplementation have been the subject of much research. Yet, no clear evidence has been presented to ascertain whether oral probiotics are effective in treating and preventing vaginal diseases. However, when there is a regular recurrence of disease as in RV, taking these probiotics may help prevent it. There have also been studies on the therapeutic efficacy of vaginal microbiome transplantation in women with serious and repetitive bacterial vaginosis (Lev-Sagie et al., 2019). In the future, vaginal microbiome transplantation may be considered a promising alternative in patients with RV whose symptoms are not improved by universal antibiotics treatment.

In conclusion, changes in species abundance and microbial diversity in the vagina were strongly associated with RV in this study. In particular, the abundance and diversity of bacterial taxa were significantly higher in women with gynecological diseases. This study provides evidence for further investigation into the vaginal microbiota of RV patients, which can aid in the development of appropriate treatment guidelines and personalized treatment for gynecological diseases.

## Data Availability Statement

The datasets presented in this study can be found in online repositories. The names of the repository/repositories and accession number(s) can be found at: EBI Metagenomics with accession PRJEB50183.

## Ethics Statement

The studies involving human participants were reviewed and approved by the Catholic University of Korea, Bucheon St. Mary’s Hospital Institutional Review Board (HC19TESI0084). The patients/participants provided their written informed consent to participate in this study.

## Author Contributions

MJK, SL, and MK conceived the idea and designed the study. MJK and MYK collected samples and analyzed data. MJK wrote the main manuscript. SL prepared Figures and Tables, including Supplementary Materials. MK reviewed and supervised the manuscript. All authors contributed to the article and approved the submitted version.

## Funding

This work was supported by the Institute of Clinical Medicine Research of Bucheon St. Mary’s Hospital, Research Fund, 2020.

## Conflict of Interest

The authors declare that the research was conducted in the absence of any commercial or financial relationships that could be construed as a potential conflict of interest.

## Publisher’s Note

All claims expressed in this article are solely those of the authors and do not necessarily represent those of their affiliated organizations, or those of the publisher, the editors and the reviewers. Any product that may be evaluated in this article, or claim that may be made by its manufacturer, is not guaranteed or endorsed by the publisher.

## Footnotes

^1^ https://www.ezbiocloud.net/apps

## Abbreviations

RV: Recurrent vaginitis
