# Supplementary material for: Clinical Significance of Composition and Functional Diversity of the Vaginal Microbiome in Recurrent Vaginitis
Source: Front Microbiol. 2022 Feb 18;13:851670. doi: 10.3389/fmicb.2022.851670 (PMC8895140; doi:10.3389/fmicb.2022.851670)
Supplement: Supplementary file 1 [file Data_Sheet_1.PDF]

Supplementary Table 1. The microbiome taxonomic composition according to the vaginal community state type (CST) in recurrent vaginitis (n=40).

CST	Case	Microbiome taxonomic composition
I	36	***Lactobacillus crispatus* (90%), *Lactobacillus iners* (10%)**
	37	***L. crispatus* (97%)**, *Ureaplasma urealyticum* (3%)
	32	***L. crispatus* (100%)**
	34	***L. crispatus* (47%),** *Gardnerella vaginalis* (44%), *Streptococcus agalactiae* (5%), *Veillonella montpellierensis* (4%)
	15	***L. crispatus (35%)***, *Prevotella oris* (21%), *Bifidobacterium dentium* (17%), *Prevotella bivia* (6%), *Dialister propionicifaciens* (4%), *Actinomyces neuii* group (3%), *Finegoldia magna* (2%), *Atopobium rimae* group (1%), *Atopobium parvulum* (1%), HE978624_s (1%), *Peptoniphilus harei* group (1%), *Peptoniphilus coxii* (1%), *Veillonella atypica* (1%)
II	29	***Lactobacillus gasseri* group (76%)**, *Atopobium vaginae* (23%)
	26	***L. gasseri* (86%)**, *Bifidobacterium scardovii* (13%)
III	13	***L. iners* (85%), *L. crispatus* (8%), *Lactobacillus jensenii* (5%)**
	28	***L. iners* (94%),** *U. urealyticum* (5%)
	11	***L. iners* (100%)**
	12	***L. iners* (100%)**
	24	***L. iners* (100%)**
	8	***L. iners* (98%)**
	20	***L. iners* (97%)**
	17	***L. iners* (71%)**, *G. vaginalis* (19%), ***L. crispatus* (8%)**, ***Lactobacillus reuteri*** (1%)
IV	25	*S. agalactiae* (99%)
	2	*S. agalactiae* (90%), ***Lactobacillus ruminis*** (6%), *Enterobacteriaceae* group (2%), AF385567_s (1%)
	19	*G. vaginalis* (89%), ***L. gasseri* group (11%)**
	21	*G. vaginalis* (100%)
	3	*G. vaginalis* (96%), *Mycoplasma hominis* (1%), *U. urealyticum* (1%)
	38	*G. vaginalis* (82%), ***L. iners* (18%)**
	35	*G. vaginalis* (83%), ***L. iners* (6%), *L. gasseri* (4%)**, *A. vaginae* (4%), *U. urealyticum* (2%)
	1	*G. vaginalis* (79%), HE978624_s (6%), *U. urealyticum* (5%), *Prevotella timonensis* (5%), ***L. crispatus* (2%)**
	30	*A. vaginae* (76%), *G. vaginalis* (23%)
	23	*A. vaginae* (92%). *Streptococcus anginosus* group (5%), *Aerococcus christensenii* (1%)
	18	*Gemella asaccharolytica* (47%), *P. bivia* (23%), *S. anginosus* group (20%), *Dialister micraerophilus* (8%), *Escherichia coli* group (1%)
	7	*S. anginosus* group (73%), *Enterobacteriaceae* group (13%), FWNZ_s (5%), *Anaerococcus hydrogenalis* (2%)
	33	*G. vaginalis* (47%), ***L. iners* (23%)**, *A. vaginae* (23%), ADGP_s (6%)
	16	*G. vaginalis* (56%), *A. vaginae* (19%), ***L. iners* (16%)**, *A. christensenii* (7%), *Chlamydia trachomatis* (1%)
	39	*G. vaginalis* (49%), *A. vaginae* (33%), *Prevotella amnii* (9%), ADGP_s (6%), KQ959671_s (1%)
	4	*G. vaginalis* (52%), *A. vaginae* (25%), *P. timonensis* (8%), KQ959578_s group (5%), ADGP_s (3%), *Mobiluncus curtisii* (2%), KQ959671_s (1%), KQ960846_s (1%)
	22	*G. vaginalis* (32%), ***L. iners* (28%)**, *A. christensenii* (10%), *A. vaginae* (10%), *V. montpellierensis* (7%), *G. asaccharolytica* (5%), *P. bivia* (4%), *U. urealyticum* (1%)
	14	*G. vaginalis* (45%), *Leptotrichia amnionii* (35%), KQ959671_s (8%), *P. timonensis* (6%), ADGP_s (2%), *Porphyromonas asaccharolytica* (1%)
	5	*G. vaginalis* (31%), *L. amnionii* (22%), *A. vaginae* (20%), KQ959578_s group (9%), JRNC_s (7%), AFUG_s (2%), *P. timonensis* (2%), KQ959671_s (2%)

	6	*L. amnionii* (39%), *Sneathia sanguinegens* (12%), *P. asaccharolytica* (11%), *P. timonensis* (8%), *A. vaginae* (5%), *Prevotella disiens* (4%), *G. vaginalis* (3%), *M. curtisii* (3%), KQ959647_s (2%), *Anaerococcus prevotii* group (1%), *Mageeibacillus indolicus* (1%), *Porphyromonas uenonis* group (1%)
	31	*Anaerococcus*_uc (17%), HE978624_s (16%), *P. harei* group (14%), *Corynebacterium pyruviciproducens* (8%), *G. vaginalis* (7%), *Rhodococcus erythropolis* group (6%), *P. bivia* (5), AJ279038_s (3%), *Bradyrhizobium japonicum* group (2%), *A. vaginae* (2%)
V	27	***L. jensenii* (97%)**, *P. bivia* (2%)
Mixed	10	***L. jensenii* (28%)**, *A. vaginae* (26%), *G. vaginalis* (24%), ***L. crispatus* (20%)**
	40	***L. crispatus* (66%), *L. iners* (34%)**
	9	***L. iners* (58%), *L. crispatus* (38%), *L. jensenii* (2%)**

Lactobacillus species were shown in bold.
